# Effects of Replacing Ensiled-Alfalfa with Fresh-Alfalfa on Dynamic Fermentation Characteristics, Chemical Compositions, and Protein Fractions in Fermented Total Mixed Ration with Different Additives

**DOI:** 10.3390/ani11020572

**Published:** 2021-02-22

**Authors:** Run Gao, Ying Luo, Shengyang Xu, Musen Wang, Zhiqiang Sun, Lei Wang, Zhu Yu

**Affiliations:** 1 College of Grassland Science and Technology, China Agricultural University, Beijing 100193, China; 15847113529@163.com (R.G.); luoying7489@163.com (Y.L.); sy20193040640@cau.edu.cn (S.X.); sunzhiqiang@cau.edu.cn (Z.S.); wanglei938210@163.com (L.W.); 2 School of Life Sciences, Lanzhou University, Lanzhou 730000, China; wangms@lzu.edu.cn

**Keywords:** fresh-alfalfa, *Lactobacillus plantarum*, fermented total mixed ratio, fermentation profile, protein degradation

## Abstract

**Simple Summary:**

Alfalfa (*Medicago sativa*) is commonly used as a high-quality protein source in fermented total mixed ration (FTMR) for ruminants. This study evaluated the fermentation characteristics, chemical compositions, and protein fractions of FTMR using fresh-alfalfa as the main ingredients replacing ensiled-alfalfa. The results showed that fresh-alfalfa FTMR exhibited a similar pH, propionic acid content and neutral detergent fiber, nonprotein, and variable to slow protein and indigestible protein levels in comparison to ensiled-alfalfa FTMR. Therefore, the use of fresh-alfalfa as a main ingredient in FTMR is promising.

**Abstract:**

Alfalfa (*Medicago sativa*) is one of the high protein ingredients of fermented total mixed ration (FTMR). Additionally, FTMR is widely used to satisfy the nutrition requirements of animals. This study was conducted to confirm the fermentation characteristics, chemical compositions and protein fractions changes when replacing ensiled-alfalfa with fresh-alfalfa in FTMR with additives. Three additives were separately applied to fresh-alfalfa total mixed ration (TMR) and ensiled-alfalfa TMR, including molasses (MOL), *Lactobacillus plantarum* (LP) and MOL plus LP (MOL+LP). The same volume of distilled water was sprayed onto the prepared TMR as performed for the control (CK). Each treatment included 18 repetitions and opened 3 repetitions at each fermenting day (1, 3, 7, 15, 30 and 60 d). The results showed that fresh-alfalfa FTMR (F-FTMR) exhibited slight changes in the fermentation characteristics during the first 7 d and showed similar trends in terms of the pH and organic acids content to ensiled-alfalfa FTMR (E-FTMR). The lactic acid contents of F-FTMR were significantly lower than those of E-FTMR at 60 d fermentation and the ammonia nitrogen contents were lower than E-FTMR during the entire fermenting period. The crude protein of the F-FTMR was enhanced after 60 d of fermenting. F-FTMR supplemented with MOL+LP exhibited a lower nonprotein nitrogen content, variable to slow protein and indigestible protein contents, and higher fast degradable protein and true protein degraded intermediately contents at 60 d fermenting, indicating that it effectively inhibited protein degradation.

## 1. Introduction

Recently, total mixed ration (TMR) have been widely utilized for livestock because they can provide balanced nutrition that satisfies ruminants’ nutritional requirements [1]. In particular, the utilization of fresh-forage TMR in dairy cows is becoming popular again [2,3]. However, the proliferation of molds, yeasts, and bacteria easily occurs due to the presence of sufficient nutrients and moisture content in TMR during long-distance transportation, resulting in the nutritional deterioration of TMR [4,5]. Therefore, most farmers and companies use stretch film sealing TMR as fermented TMR (FTMR), which can effectively inhibit TMR deterioration and save nutrients for a longer period of time [6]. Compared with TMR, FTMR preparation is more efficient and less time-consuming, which can greatly economize labor and resources [7]. At the same time, FTMR can be transported over a long-distance, and can be purchased and utilized by some small-scale farms in the absence of TMR mechanical equipment [7]. Moreover, FTMR can improve feeding levels and promote the economic development of animal husbandry [8]. FTMR is very common in dairy cows, especially ensiled-alfalfa (*Medicago sativa*) FTMR. Generally, it is believed that ensiled-alfalfa FTMR can improve the milk yield and quality of dairy cows [9,10].

However, it is rare to ferment TMR with fresh-alfalfa as one of the ingredients. Due to the high-quality of alfalfa [11], fresh-alfalfa may improve the quality of FTMR and improve the feeding level of ruminants. Compared with ensiled-alfalfa TMR (E-FTMR), fresh-alfalfa FTMR preparation takes less time, subsequently shortening the production cycle of FTMR and potentially reducing the costs of production [2].

The true protein of alfalfa is degraded to nonprotein nitrogen (NPN), which accounts for up to 80% of total nitrogen (TN) [12]. However, problems may also exist for fresh-alfalfa FTMR (F-FTMR). In addition, the chemical compositions may differ [7], and extensive proteolysis may still occur during fermentation. It is necessary to evaluate the difference between F-FTMR and E-FTMR and the quality of F-FTMR after different fermenting durations.

Therefore, the objective of this experiment was to determine the effects of replacing ensiled-alfalfa with fresh-alfalfa in FTMR on the fermentation characteristics, chemical compositions and protein fractions of FTMR with additives under different fermenting durations.

## 2. Materials and Methods

### 2.1. Fermented TMR Preparation

Alfalfa (Gannong No. 4) in this study was harvested at the early flowering stage of the third cutting in September 2018 (Yanchi, Ningxia, China). Part of the alfalfa was harvested by hand with a sickle, leaving 5-cm stubble. Then, alfalfa was immediately chopped into 1- to 2-cm pieces with a chopper (9Z-0.4, Jinniu Machinery Factory, Rongyang, Henan, China). The fresh-alfalfa was mixed with corn silage, oat hay and other concentrates as the ratio F-FTMR showed in Table 1. Additives were dissolved in distilled water in the present study, including molasses (MOL, 2% fresh weight (FW)), *Lactobacillus plantarum* (LP, 1 × 10^6^ cfu/g FW) and MOL (2% FW) plus LP (1 × 10^6^ cfu/g FW) (MOL+LP). The same volume of distilled water was sprayed onto the prepared TMR as a control (CK). Then, 300 g of the prepared TMR diets was packed into polyethylene bags, and the bags were sealed with a vacuum sealing machine and stored at room temperature until opening. The three replications, four treatments and six fermenting days resulted in a total of 72 bags. Three replications of the 0 d sample were tested without sealing. In addition, another part of alfalfa was wilted to a 55% moisture content, chopped and wrapped with stretch film. After having been stored at room temperature for 60 d, the wrapped alfalfa was opened and mixed with corn silage, oat hay and concentrates as the ratio E-FTMR showed in Table 1. The TMR diets based on ensiled-alfalfa were also designed with four treatments as described above, vacuumed sealed and stored at room temperature until opening. In general, 72 bags were also prepared (three replications, four treatments and six fermenting days). In addition, three replications of the 0 d samples were tested without sealing. After 1, 3, 7, 15, 30 and 60 d fermenting, 12 bags (CK, MOL, LP and MOL+LP) of F-FTMR and 12 bags (CK, MOL, LP and MOL+LP) of E-FTMR were opened and detected, respectively.

The corn silage was stored in silage trenches for approximately one year. The MOL was purchased from Rongxin Chemical Industry (Jinan, Shandong, China). The strain of *L. plantarum* was isolated from a solution of sheepskin by the silage laboratory of China Agricultural University and identified as *L. plantarum* by 16S rRNA gene sequencing [13].

### 2.2. Fermentation Characteristics, Chemical Compositions, and Protein Fractions

At each opening, twenty grams of each sample was mixed with 180 mL of distilled water. The mixture was homogenized with a blender for 60 s and successively filtered with 4-layer nylon gauze and qualitative filter paper [14]. The pH was measured immediately using a pH meter (Five Easy Plus FE28, Mettler Toledo Co., Ltd., Shanghai, China). The ammonia nitrogen (AN) concentration was determined according to the method of Broderick and Kang [15], and the concentrations of organic acids, including lactic acid (LA), acetic acid (AA), propionic acid (PA) and butyric acid (BA), were analyzed via high-performance liquid chromatography (HPLC) as previously described [16].

TMR samples obtained before and after fermentation were dried in an oven (GZX-9140MBE, Shanghai Boxun Co. Ltd., Shanghai, China) at 65 °C for 48 h for DM measurement. The dry samples were ground in a hammer mill (FZ102, Test Instrument, Tianjin, China) so that they passed through a 1-mm sieve for further chemical analysis. The crude protein (CP) was analyzed with an automatic Kjeldahl nitrogen apparatus (Kjeltec2300 Auto-Analyzer, FOSS Analytical AB, Hoganas, Sweden) according to the AOAC [17]. The neutral detergent fiber (aNDF) and acid detergent fiber (ADF) contents were determined as described by Van Soest et al. [18] with an Ankom fiber analyzer (A2000I, Ankom Technology, Macedon, NY, USA). Sodium sulfite and thermostable α-amylase were used in the aNDF analysis, and the results are expressed on a DM basis. The water-soluble carbohydrates (WSC) were determined using the anthrone method of Thomas [19]. NPN, soluble protein (SOLP), neutral detergent insoluble protein (NDIP) and acid detergent insoluble protein (ADIP) were determined to calculate the protein fractions [20].

### 2.3. Calculations

Protein fractions, including PA_NPN_ (NPN), PB_1_ (true protein degraded rapidly), PB_2_ (true protein degraded intermediately), PB_3_ (true protein degraded slowly), and PC (bound true protein), were calculated according to the Cornell Net Carbohydrate and Protein System (CNCPS) using the following formulas:

PA_NPN_ (%CP) = NPN (%CP), PB_1_ (%CP) = SOLP (%CP) − PA_NPN_ (%CP), PB_2_ (%CP) = 100 − PA_NPN_ (%CP) − PB_1_ (%CP) − PB_3_ (%CP) − PC (%CP), PB_3_ (%CP) = NDIP (%CP) − ADIP (%CP), PC (%CP) = ADIP (%CP).

The relative feed value (RFV) index was estimated as follows [21]:

RFV = (DDM × DMI)/1.29, DDM = Digestible dry matter = 88.9 − (0.779 × %ADF), DMI = Dry matter intake (% of BW) = 120/(%NDF).

The total digestible nutrient (TDN) content was estimated as following the method of Holland et al. [22]:

TDN(%) = 88.9 − (0.79 × ADF).

### 2.4. Statistical Analyses

The experiment followed a completely randomized design with a 2 × 3 × 4 × 6 (2 styles, 3 repetitions, 4 treatments, and 6 opening days) factorial arrangement. The ANOVA procedure in SAS 9.4 (version 9.4, SAS Institute Inc., Cary, NC, USA) was used to test the effects of treatments during each fermenting period. Post hoc mean comparisons were performed with Duncan’s multiple range test. To assess the chemical profiles and fermentation quality, repeated measurements were obtained during the fermentation period (1, 3, 7, 15, 30, and 60 d after fermentation) within each experimental treatment. The protein fractions of the diets were tested before and after fermentation. The GLM procedure in SAS was used to test the effects of the fermenting style and additive treatments and their interactions for each parameter. Significance was declared at *p* ≤ 0.05.

## 3. Results

### 3.1. Chemical Compositions of Fresh-Alfalfa, Ensiled-Alfalfa, and Their Mixture before Fermenting

The ingredients of the F-FTMR and E-FTMR are listed in Table 1. The chemical compositions of each ingredient were determined three times and are listed in Table 2. The chemical compositions and protein fractions of the two styles of FTMR before fermentation are presented in Table 3 and Table 4.

### 3.2. Fermentation Characteristics of TMR after Fermentation

#### 3.2.1. Fresh-Alfalfa TMR after Fermentation

During the fermentation of the F-FTMR, pH exhibited a rapid decrease during the first 15 d of fermentation. After 15 d, the pH tended to be stable until the end of fermenting (Table 5). At 3, 7, 15, and 30 d fermenting, significant differences were noted among the four treatments. In particular, the pH of LP treatment decreased more rapidly and exhibited a lower pH from 7 to 30 d than the other treatments. There were no differences among the treatments on the first and last days of fermenting. It was indicated that a long period of storage could lead to a similar pH of TMR with or without additives.

The LA concentrations of all treatments were different during the entire fermenting period. The LA concentrations of F-FTMR increased rapidly during the first 15 d of fermenting and increased slowly until d 60 (Table 5). The LA concentration of MOL+LP treatment continued to increase during the first 30 d and became stable towards the end of fermentation. Additionally, 3 d after fermentation, the LA concentrations were differed among the four treatments, the treatments LP and MOL+LP exhibited higher LA content than other treatments.

The AA concentration exhibited no difference among the additives at each fermenting day. However, whilst the fermenting time exhibited a significant effect on the AA concentration of CK, MOL, and LP treatments (Table 5), no significant effect was found on MOL+LP treatment.

The PA concentration also exhibited no difference among the four treatments at each fermenting day (Table 5). The fermenting period had a significant effect on all additive treatments during the entire fermenting period.

No BA was detected in the F-FTMR.

The AN content of all treatments increased gradually from the beginning to 30 d of fermenting and decreased slightly at 60 d. In addition, the AN content of all treatments exhibited a significant effect at 15 and 30 d fermenting (Table 5).

#### 3.2.2. Ensiled-Alfalfa TMR after Fermentation

Additives had no significant effect on the pH at each fermenting day of the E-FTMR, except on d 15 of fermenting. The pH of all additive treatments decreased significantly during the fermenting period. The pH decreased rapidly from 7 to 30 d fermenting and tended to be stable from 30 d to the end of fermenting (Table 5). All downward trends were similar, regardless of additives. No differences were noted among the additive treatments during each fermenting period, except on d 15. This finding suggests that the additives exhibited no effect on the E-FTMR. Therefore, the E-FTMR could be produced with no additives.

The LA concentration of E-FTMR exhibited significant variation during the entire fermenting period. During the entire fermenting period, the LA concentration in all treatments displayed a steady increasing trend. The LA concentration of CK increased more rapidly than the other treatments from 15 to 30 d of fermenting. Additive treatments exhibited a significant effect at 3, 30, and 60 d of fermenting. In particular, on the 30 d of fermenting, CK and MOL yielded a greater LA concentration compared with the other treatments (Table 5).

The AA content of all treatments increased gradually during the fermenting period. The AA concentration exhibited a significant difference at 7 and 60 d of fermenting, and the CK treatment exhibited a higher AA content compared with other treatments at 30 and 60 d (Table 5).

The four treatments also exhibited a significant effect on the PA concentration at each fermenting time except for 30 d, and the PA concentration of four the treatments differed during the fermenting period (Table 5). The treatments with MOL or MOL+LP exhibited higher PA concentrations compared with the other treatments at 30 and 60 d fermenting.

No BA was detected in the E-FTMR.

The AN content increased slowly during the first 3 d, decreased slightly from 3 to 15 d of fermenting, and then remained stable until the end of fermenting. On 1, 30, and 60 d of the fermentation period, the AN content of the four treatments exhibited significant difference (Table 5).

#### 3.2.3. Fermentation Quality Comparison of Fresh-Alfalfa and Ensiled-Alfalfa TMR after Fermentation

The significance for the fermentation profile variables of fresh-alfalfa and ensiled-alfalfa FTMR treated with different additives at each ensiling day were showed in Table 6. The pH of the two styles of FTMR exhibited similar decreasing trends. Specifically, the pH of the two styles of FTMR reduced from approximately 4.1 to 4.9 and became stable from 30 to 60 d of fermentation. However, the pH of E-FTMR exhibited minimal changes during the first 7 d of fermenting and began to decrease thereafter. The trend of the LA content was opposite to that of the pH, exhibiting an increasing trend after fermentation. The LA contents in the F-FTMR treatments increased rapidly during the first 15 d of fermenting, whereas those in the E-FTMR increased slowly during the first 15 d of fermenting. The LA concentration in the E-FTMR was significantly higher than that in the F-FTMR at the end of fermenting (Table 6). The AA content of the F-FTMR increased quickly during the first 3 d of fermenting, but that of the E-FTMR declined slightly. Then, the AA content increased gradually during the remaining fermenting duration, and only the MOL and LP treatments of the F-FTMR exhibited slightly decreasing trends at d 30 of fermenting. The PA content of the two styles of FTMR increased gradually, which was similar to the trend of the AA content during fermentation. No BA content was detected in the F-FTMR and the E-FTMR. The variation trends of the AN content differed between the two styles of FTMR. Specifically, the AN content in the F-FTMR exhibited a gradually increasing trend from the beginning to 30 d of fermenting and then showed few changes. However, the AN content in the E-FTMR exhibited relatively stable changes during the fermentation duration.

### 3.3. Chemical Compositions of TMR after Fermentation

#### 3.3.1. Fresh-Alfalfa TMR after Fermenting

The chemical composition of the F-FTMR after 60 d of fermentation is shown in Table 7. The DM content of the F-FTMR reduced significantly after 60 d of fermentation. The CK treatment exhibited a lower DM content than other treatments. The additives had a higher DM content than those observed in CK (Table 7). The CP content of F-FTMR increased after 60 d of fermentation. Additives exhibited no effect on the CP content after 60 d of fermentation (Table 7). Additive supplementation had no effect on the aNDF content of the F-FTMR. Overall, the aNDF content after 60 d of fermentation was higher than that before fermentation (Table 7). Additives had no effect on the ADF content of the F-FTMR, and exhibited no effect before and after fermentation. The ADF content of LP treatment was lower compared with other treatments. Overall, the content of ADF before fermentation was higher than that after 60 d of fermenting except for the treatment MOL+LP (Table 7). The WSC content of the F-FTMR reduced significantly after 60 d fermentation. The WSC content exhibited no difference after 60 d of fermentation (Table 7). The TDN content of the F-FTMR exhibited no difference before and after 60 d of fermentation, and the treatments CK, MOL and LP increased slightly (Table 7). The RFV content of the F-FTMR reduced after 60 d of fermentation except for the treatment MOL. The additives exerted no difference on the RFV content after 60 d of fermentation (Table 7).

#### 3.3.2. Ensiled-Alfalfa TMR after Fermenting

Compared with that before fermentation, the DM content of the E-FTMR was increased after 60 d of fermentation (Table 7). The use of additives increased the DM content of the E-FTMR. Additionally, the CK treatment exhibited a lower DM content after 60 d of fermenting. The CP content of E-FTMR exhibited a decreasing trend compared with the TMR before fermentation and the treatment CK; in particular, the additive treatments showed a lower CP content. The aNDF content of E-FTMR exhibited fewer decreasing trend, and the treatment MOL+LP exhibited a higher aNDF content than other treatments. The ADF content of the E-FTMR exhibited no difference among all treatments before and after fermentation (Table 7). The WSC content of the E-FTMR reduced significantly after 60 d of fermentation. The WSC content exhibited no difference among all treatments after 60 d of fermentation (Table 7). The TDN content of the E-FTMR exhibited no difference before and after 60 d of fermentation (Table 7), the treatments MOL and LP increased slightly. The RFV content of the E-FTMR increased after 60 d of fermentation, and the treatment MOL exhibited higher RFV content (Table 7).

#### 3.3.3. Chemical Profile Comparison of Fresh-Alfalfa and Ensiled-Alfalfa TMR after Fermentation

The DM content of F-FTMR before fermentation were significant lower than E-FTMR. The DM content of the F-FTMR declined after 60 d of fermentation and the DM content of the E-FTMR increased after 60 d of fermentation. The CP content exhibited a significant difference between the two styles of FTMR. The content of CP in F-FTMR was increased after 60 d of fermentation, and the CP content of E-FTMR decreased after 60 d of fermentation. The aNDF content of the F-FTMR increased slightly, whereas that of the E-FTMR exhibited decreasing trends. The changes of ADF content of F-FTMR and E-FTMR exhibited no difference before and after fermentation. The WSC content of F-FTMR was higher than E-FTMR before fermentation. After 60 d of fermentation, both styles of FTMR exhibited no significant difference. The TDN content of the two styles of FTMR displayed no difference and the treatments MOL and LP increased in both F-FTMR and E-FTMR. The RFV content of the F-FTMR was higher than that of the E-FTMR before fermentation. After 60 d of fermentation, the treatment E-FTMR exhibited the highest RFV content.

### 3.4. Protein Fractions (CNCPS) of TMR before and after Fermentation

**Figure 1:**
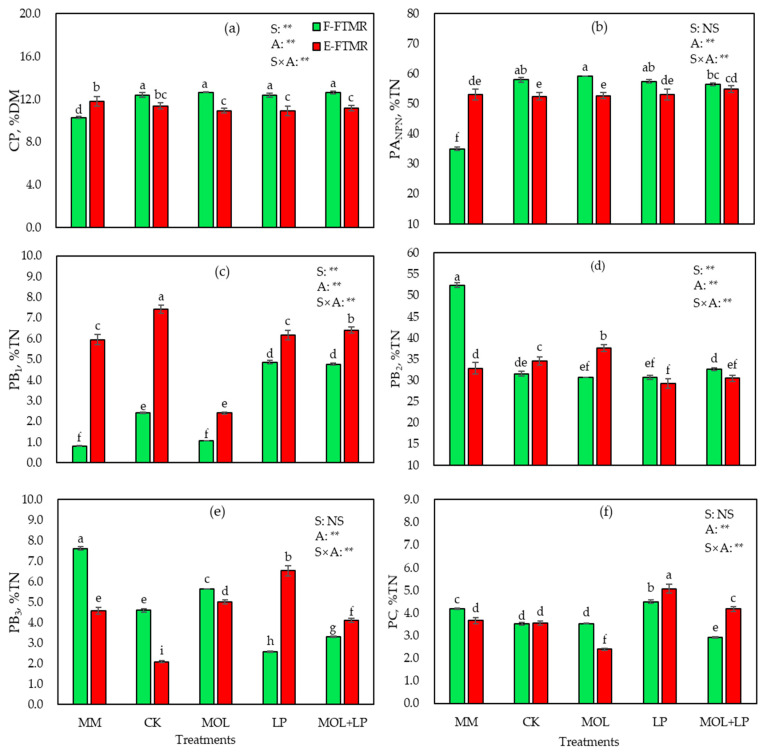
The protein fractions (CNCPS) of fresh-alfalfa FTMR and ensiled-alfalfa FTMR before fermentation and at 60 d fermentation in (**a**) CP, crude protein; (**b**) PA_NPN_, not applicable protein; (**c**) PB_1_, fast degradable protein; (**d**) PB_2_, variable protein; (**e**) PB_3_, variable to slow protein; and (**f**) PC, indigestible protein (bars indicate standard error of the means). F-FTMR represents the FTMR based on fresh-alfalfa. E-FTMR represents the FTMR based on ensiled-alfalfa. MOL, LP, and MOL+LP represent the addition of molasses, *Lactobacillus plantarum* and molasses mixed with *L. plantarum*, respectively. CK represents the addition of an equal amount of distilled water to the diets. DM represents dry matter, TN represents total nitrogen, and MM represents the mixed material before fermenting. A denotes significance of the additive; S denotes significance of two fermented style of FTMR; A×S denotes significance of the interaction between the additive and style. The asterisks (** *p* ≤ 0.01) indicate the significance of FTMR styles and additives and their interaction effects, and the difference at the specified ensiling day. NS, no significance. ^a–i^ Means significant difference among MM and all treatments at 60 d of fermentation (*p* < 0.05).

The CP content and protein fractions, which were defined by the CNCPS, before fermentation and after 60 d of fermentation are shown in Figure 1. The content of CP in F-FTMR increased after 60 d of fermenting, and the four treatments had no significant effects (Figure 1a). However, the CP content of the E-FTMR after 60 d of fermenting was slightly lower than that of the mixed material before fermentation and F-FTMR at 60 d of fermenting, and the additives had no effect on the CP content.

The PA_NPN_ proportion of the F-FTMR increased after 60 d of fermenting compared with mixed material before fermentation, and the E-FTMR displayed fewer changes compared with mixed material before fermentation (Figure 1b). Additionally, the PA_NPN_ proportion of the F-FTMR was higher than that of the E-FTMR after 60 d of fermenting. Furthermore, the PA_NPN_ proportion had strong effects on the F-FTMR, but fewer effects on the E-FTMR. The F-FTMR supplemented with MOL+LP exhibited a lower PA_NPN_ proportion compared with MOL treatment, and exhibited a similar proportion to mixed material and MOL+LP of E-FTMR.

After 60 d of fermenting, the PB_1_ proportion of F-FTMR increased compared with the mixed material before fermentation, but MOL yielded a similar PB_1_ proportion with mixed material before fermentation (Figure 1c). The LP and MOL+LP treatments exhibited a higher PB_1_ proportion than mixed material, CK and MOL treatments. Moreover, the PB_1_ proportion in the E-FTMR increased at 60 d of fermenting, except for MOL treatments. The PB_1_ proportion of CK and LP and MOL+LP in E-FTMR was higher, and LP yielded a similar PB_1_ proportion with the mixed material. The PB_1_ proportion of MOL treatment was the lowest.

The PB_2_ proportion of the F-FTMR declined dramatically after 60 d of fermenting (Figure 1d). However, CK and MOL treatments of the E-FTMR exhibited a higher PB_2_ proportion than mixed material and other treatments. Moreover, the treatment of E-FTMR with MOL yielded a significantly greater PB_2_ proportion. The PB_2_ proportions of LP in E-FTMR were lower than others.

The proportion of PB_3_ differed between the two styles of FTMR at 60 d of fermenting. The PB_3_ proportion of F-FTMR exhibited a strong decreasing trend at 60 d of fermenting (Figure 1e). F-FTMR treated with MOL had a higher proportion compared with other treatments, but the PB_3_ proportion in the LP treatment was reduced significantly compared with other treatments. However, the proportion in the CK of the E-FTMR was lower than that in the mixed material and other treatments, and the LP treatment exhibited the highest PB_3_ proportion after fermentation.

The PC proportion of F-FTMR was reduced compared with that of the mixed material, except for LP treatment, in which it was strongly increased (Figure 1f). The PC proportion of MOL in F-FTMR was similar to that in the CK treatment, and LP treatment was significantly greater than mixed material and other treatments. However, the PC proportion of MOL+LP treatment in F-FTMR was lower than in other treatments and mixed material. However, the proportion in the MOL treatment of the E-FTMR was lower than in other treatments. The PC proportions in the LP treatment were higher than those in the other treatment and mixed material in both of the FTMR styles.

## 4. Discussion

### 4.1. Fermentation Characteristics of TMR after Fermentation

The pH is a key factor affecting the success and quality of silages [23]. The pH of the two styles of FTMR was reduced from approximately 4.1 to 4.9 in our study, which is similar to what was reported by Chen et al. [3], where the pH of all treatments TMR silage were reduced after 45 d fermenting, suggesting excellent fermentation. In the present study, the pH of the F-FTMR displayed a rapidly decreasing trend, whereas that of the E-FTMR exhibited slight changes at 7 d of fermentation. The pH of F-FTMR was consistent with the results of Zhao et al. (2018) [24], who found that LP caused a rapid pH decline at the initial stage of fermentation, but the E-FTMR exhibited few changes at the beginning of fermentation. One possible reason for this difference is that the fresh-alfalfa had sufficient moisture and substrates to react with other ingredients. In contrast to the E-FTMR, the ensiled-alfalfa had already ensiled for 60 d, and the substrates and lactic acid bacteria (LAB) were completely fermented. The addition of LP and MOL+LP resulted in a lower pH and AN concentration in the F-FTMR. This finding is consistent with the finding of Sun et al. (2009) [25], who reported that LAB were more effective in ensiling forages. In the present study, the LP and MOL additives had a synergistic effect on the AN concentration in the F-FTMR. An AN content of less than 10% TN represents well-preserved silages and lower proteolysis in silages [3,26]. The F-FTMR (0.07–3.67% TN) exhibited a lower AN content throughout the fermenting duration than the E-FTMR (5.14–7.27% TN) in our study. The AN proportion that accounted for TN was lower than that in crop silage (10–20% TN) in all treatments in our study, possibly because deamination of TMR may be inhibited compared with that of crop silage [5,27]. The application of MOL, LP, and MOL+LP had no effects on the pH in the two styles of FTMR at 60 d of fermenting. The effect of MOL treatment was similar to that reported by Chen et al. [3], where MOL had no effect on the pH of ensiled TMR at 45 d of fermenting compared with the control, but LP had the opposite effect at 60 d fermenting in our study. Additionally, Chen et al. also reported that adding LAB resulted in a lower AA content and AN/TN ratio compared with those in the control, which is consistent with the effect of adding LP in our study. However, no BA was observed in our study, which was inconsistent with the finding of the previous study, in which the BA content increased with MOL additive. Silva et al. [28] reported that inoculants resulted in a similar pH of ensiled-alfalfa at 56 d, and these values were lower than those in the CK. In our study, all additives resulted in a lower pH compared with those in the CK at 60 d of fermenting.

TMR silages contain high organic acid contents [8,29]. The LA content of the two styles of FTMR increased after fermentation. The content in the F-FTMR treatments increased more rapidly than E-FTMR during the first 15 d of fermenting. The LA concentration in the E-FTMR was higher than that in the F-FTMR at the end of fermenting. Similar to the findings of previous studies, the range of the LA concentration of TMR was 4–10% DM at the end of fermenting in our study, and this finding may be attributed to the different ingredients, seasons, and fermenting methods [5,8,29,30,31]. LP application resulted in lower LA and AA contents compared with those in the CK at 60 d of fermenting in our study. This result is in contrast to that observed by Chen et al. [3] in which the addition of LAB increased the LA content, which may be attributed to the low WSC concentration of alfalfa used in TMR silages [32]. The addition of LP or MOL+LP resulted in a lower LA concentration compared with that in the CK, and MOL applied at 60 d of fermenting, especially to the E-FTMR, resulted in a notable difference. This difference may result in the presence of clostridia, which can consume LA [33].

In our study, the AA content of the F-FTMR increased quickly during the first 3 d of fermenting, but that of the E-FTMR declined slightly. Thereafter, the AA contents of the two styles of FTMR increased gradually during the remaining fermenting period. Only the MOL and LP treatments of the F-FTMR resulted in decreases on d 30 after fermentation, and LP treatment resulted in the lowest AA content at 30 and 60 d fermenting. One possible reason for this is that alfalfa accounted for the majority of the F-FTMR, and was similar to alfalfa silage. Silva et al. [28] reported that adding *Pediococcus acidilactici* and *Pediococcus pentosaceus* resulted in lower AA concentrations than the control at 3 and 28 d of fermentation of alfalfa silage, and adding *P. pentosaceus* resulted in the lowest AA content at 56 d. An AA concentration lower than 2% DM represents well-fermented silage [28], and the AA contents of the two styles of FTMR were lower than 2% DM in the present study. The PA content of the two styles of FTMR increased gradually, which was similar to the AA content during fermenting. The result was opposite to that reported by Silva et al. [28], who reported that few changes were found for the PA content in all ensiled-alfalfa during the fermentation period. However, the MOL and MOL+LP treatments had higher PA concentrations than the other treatments in both of the two styles of FTMR throughout the fermenting period. A possible reason for this may be that adding MOL not only increased the fermentative substrate of LAB, but also increased the fermentative substrate of *Clostridium* and *Propionibacterium* in FTMR. This result is similar to that of Chen et al. [3], who found that the PA content of MOL treatment was higher than that of the control and *L. plantarum*, but no differences were detected among them. No BA was found in the F-FTMR and E-FTMR in the present study. A possible reason for this is that the higher DM content of TMR was not conducive to *Clostridium* fermentation [27]. Kondo et al. [5] reported low BA in TMR silages during fermenting. Additionally, Chen et al. [3] found that TMR silage supplemented with MOL exhibited a higher BA content compared with other treatments of TMR, which is contrary to the finding of the present study.

### 4.2. Chemical Composition of TMR after Fermentation

Successful silages contain a proper DM content, a fermentable sugar concentration of 3.0–5.0%DM, and lower BC and LAB populations with greater than 1 × 10^5^ cfu g^−1^ FM [34]. It is difficult to obtain similar moisture concentrations when ensiling TMR under experimental or farm conditions [35]. The DM contents of the two styles of FTMR were 42%DM and 46%DM before fermentation in the present study, which suggests that the DM content of the TMR was beneficial for producing high-quality FTMR. The DM content influenced the rate and extent of fermentation. The DM content of the TMR was high enough to produce a high-quality FTMR, which may be well-accepted by animals [3,36]. The DM content of the F-FTMR gradually decreased by about 3.9% from the beginning to d 15 of fermenting in our study. One possible reason for this is that gases, microbial fermentation, and plant respiration of fresh-alfalfa resulted in a decrease in the DM content [27]. Additionally, a high level of production of CO_2_ increases DM loss and may decrease the DM content, as observed in ensiled-alfalfa [28]. Gases were observed for the F-FTMR, which may be related to the low DM content in our F-FTMR. This result is inconsistent with that of Kondo et al. [5], who reported no gases in any of the examined TMR silages. In our study, the DM content of the two styles of FTMR was lower in the CK treatment than in the additive treatments during the entire fermenting period. This finding is similar to the finding of Chen et al. [3], who reported that the control had lower DM contents compared with the other treatments.

The chemical composition of TMR is more important than that of fermentation products because nutrients directly affect animals and their byproducts. Chen et al. [3] reported that no notable differences were found in CP, ether extract (EE), ADF, ash, and non-fiber carbohydrates (NFC) among FTMR, but the DM, aNDF, and hemicellulose contents differed among FTMR with additives. Similarly, Kondo et al. [5] reported that the organic matter (OM), CP, EE, and NDF contents exhibited no distinct changes in TMR during the fermentation period. The aNDF content of the F-FTMR with additives was similar to that in the CK at the end of fermenting in the present study. However, for the E-FTMR, the aNDF content in the CK was similar to that in the LP treatment, higher than that in the MOL treatment, and lower than that in the MOL+LP treatment. The ADF content of the two styles of FTMR supplemented with MOL+LP was higher than that in the other treatments at the end of fermentation. The CP content of forages is one of the most important parameters for evaluating the forage nutritional value. In our study, the CP content of the F-FTMR was increased after fermentation. This result is similar to that of Kondo et al. [5], who reported that the TMR had a higher CP content at 30 d of fermenting compared with before fermentation. The CP content of the F-FTMR increased gradually during the first 15 d of fermenting and became stable during the remainder of the fermenting period. A possible reason for this may be that fresh-alfalfa in F-FTMR reacted with microorganisms and produced organic acids, water, and CO_2_ as fermenting occurred. The gases, organic acids, and water produced in the polyethylene bags escaped as they were opened. Therefore, the relative content of DM decreased gradually, but the contents of TN changed less drastically. Due to this, the relative CP contents of F-FTMR were increased, resulting in a higher CP content than that in the mixed material before fermentation. However, the ensiled-alfalfa had fermented completely before mixed with other forages in E-FTMR, so the CP content exhibited fewer decreasing.

The CP and aNDF contents of the F-FTMR increased after 60 d of fermenting, which was consistent with the finding of Kondo et al., who reported that the CP content increased after 30 d of fermentation [5]. This result is similar to that of Moselhy et al. [37], who reported that LAB inoculation increased the CP content of *Hedychium gardnerianum* silage. The possible reason for this is that the loss of gases, organic acids and water produced during the fermenting process reduced the relative content of DM, resulting in a relatively higher CP content of F-FTMR. Kondo et al. [5] observed that adding LAB resulted in a lower aNDF content compared with that in the control. This change was similar to that observed in the F-FTMR with LP addition at 60 d of fermenting. Liu et al. [38] who reported that the TDN of Italian ryegrass silage were lower than before ensiling. However, the TDN content treated MOL and LP exhibited higher TDN content in both of the FTMR. The possible reason may be that the additives could increase the TDN content of FTMR, and it requires further research.

### 4.3. Protein Fractions (CNCPS) of the TMR after Fermentation

The CNCPS is used to evaluate the protein quality and predict the rates of protein digestibility in the rumen [39]. Protein degradation during crop ensiling is thought to be caused by proteases from fresh plant material and microorganisms [27]. It has been reported that part of the true protein is degraded to NPN during the ensiling period, which accounts for approximately 80% of the TN at the opening of silos [12]. Excessive NPN content can decrease the utilization efficiency in the rumen [40]. The NPN content of alfalfa after ensiling increased [41]. It was similar to present study, all F-FTMR treatments resulted in an increasing of PA_NPN_ fraction compared with that in the mixed material before fermenting. However, the additives of LP and MOL+LP in F-FTMR resulted in a lower PA_NPN_ percentage compared with the CON and MOL. This finding is consistent with Wang et al. [42] who reported that LP could decrease the PA_NPN_ content compared with that in the control. The MOL treatment resulted in the highest PA_NPN_ proportion compared with the control and the other treatments. This finding is potentially explained by the fact that adding MOL increases the activity of plant proteases and microbial enzymes, which dominate during the protein degradation of forage silages [43]. In a previous study, protein hydrolysis was inhibited by the application of cellulase and *Lactobacillus casei*, which is similar to the finding of the present study for the application of LP and MOL+LP in relation to the low AN content and increased PB_1_ and PB_2_ proportions [44]. Guo et al. [45] reported that protein degradation was inhibited with additives during fermenting compared with than that in the control. This finding was inconsistent with the result of the present study, in which LP and MOL+LP in F-FTMR had lower AN and PA_NPN_ contents than the control at 60 d of fermenting. Protease activities are lower when grains are used as TMR ingredients because they are well-matured and un-germinated [46,47]. In the present study, there were no fresh forages but hay, silages, and grains in E-FTMR, and they were not beneficial for microorganisms and protease activities in E-FTMR diets. PA_NPN_ exhibited fewer changes in the E-FTMR after 60 d of fermenting, which may be attributed to the grains with low protease activities, low *Clostridium* and enterobacteria activities, and a high DM content in the FTMR [27,29]. Protein degradation during fermenting may be enhanced by LAB [48], which is similar to the finding of the present study, where the application of LP and MOL+LP resulted in a lower PA_NPN_ content. In the present study, the application of MOL+LP in the F-FTMR resulted in a lower AN concentration and PA_NPN_, PB_3_, and PC contents, and higher PB_1_ and PB_2_ contents compared with the other treatments, thus revealing the stronger inhibition of protein degradation. The result is similar to that of Li et al. [11], who reported that ensiled-alfalfa treated LP with sucrose resulted in lower AN, PA_NPN_, and PC contents and higher PB_2_ compared with in the control at 35 d of fermentation.

## 5. Conclusions

The present study demonstrated that additives had a minimal effect on the pH and organic acids of the two styles of FTMR. Both styles of FTMR exhibited minimal changes in the pH and organic acids during the first 7 d after fermentation. This finding indicates that FTMR can be used during the first week after fermentation. The AN content of the F-FTMR were lower than those of the E-FTMR during the fermentation period. The CP of the F-FTMR was enhanced after fermentation, whereas that of the E-FTMR decreased slightly. The application of MOL+LP effectively inhibited protein degradation in the F-FTMR. The use of fresh-alfalfa to replace ensiled-alfalfa as an ingredient in FTMR is promising.

## Figures and Tables

**Table 1:** Ingredients and proportions of the fresh-alfalfa and ensiled-alfalfa fermented total mixed ratio (FTMR).

Item	F-FTMR ^1^	E-FTMR ^2^
Ingredients Composition, % of DM		
Fresh-alfalfa	27.00	-
Ensiled-alfalfa	-	27.00
Corn silage	26.50	26.50
Oat hay	21.50	21.50
Corn grain	23.30	23.30
Soybean meal	0.50	0.50
Premixture	0.60	0.60
Sodium chloride	0.60	0.60
Total	100	100

^1^ F-FTMR represents the FTMR based on fresh-alfalfa. ^2^ E-FTMR represents the FTMR based on ensiled-alfalfa. DM, dry matter.

**Table 2:** The chemical composition of each ingredient and fresh-alfalfa and ensiled-alfalfa FTMR before fermentation.

Item ^1^	DM	CP	aNDF	ADF
	%	%DM		
Fresh-alfalfa	21.05 ± 0.57	19.44 ± 0.32	37.78 ± 0.71	28.52 ± 0.23
Ensiled-alfalfa	46.19 ± 2.32	19.49 ± 0.17	39.69 ± 1.81	30.82 ± 1.84
Corn silage	29.91 ± 1.50	8.26 ± 0.23	46.98 ± 0.49	24.35 ± 1.14
Oat hay	91.51 ± 0.03	8.18 ± 1.00	43.90 ± 1.14	23.20 ± 0.63
Corn grain	88.76 ± 0.35	9.99 ± 0.41	13.42 ± 0.97	3.85 ± 0.15
Soybean meal	91.99 ± 0.57	49.26 ± 0.34	19.94 ± 0.44	8.76 ± 0.48
Premixture	98.35 ± 0.57	0.94 ± 0.05	21.94 ± 0.75	15.09 ± 0.32

^1^ DM, dry matter; CP, crude protein; aNDF, neutral detergent fiber; ADF, acid detergent fiber.

**Table 3:** The chemical composition of fresh-alfalfa and ensiled-alfalfa FTMR before fermentation.

Item ^1^	DM	CP	aNDF	ADF	WSC	TDN	RFV
	%	%DM					
F-FTMR	41.56 ± 0.41	10.28 ± 0.06	32.78 ± 1.00	23.27 ± 0.45	5.42 ± 0.34	70.52 ± 0.36	201.26 ± 6.92
E-FTMR	46.30 ± 0.16	11.80 ± 0.27	38.36 ± 1.54	22.26 ± 1.47	2.49 ± 0.14	71.31 ± 1.16	174.31 ± 9.96

^1^ DM, dry matter; CP, crude protein; aNDF, neutral detergent fiber; ADF, acid detergent fiber; WSC, water-soluble carbohydrates; TDN, total digestible nutrient; and RFV, relative feed value. F-FTMR represents the FTMR based on fresh-alfalfa. E-FTMR represents the FTMR based on ensiled-alfalfa.

**Table 4:** The Cornell Net Carbohydrate and Protein System (CNCPS) proportion of alfalfa, fresh-alfalfa and ensiled-alfalfa FTMR before fermentation.

Item ^1^	PA_NPN_	PB_1_	PB_2_	PB_3_	PC
	%CP				
F-FTMR	34.96 ± 0.63	0.81 ± 0.01	52.43 ± 0.51	7.62 ± 0.07	4.19 ± 0.04
E-FTMR	52.97 ± 1.88	5.95 ± 0.24	32.86 ± 1.31	4.57 ± 0.18	3.66 ± 0.15

^1^ PA_NPN_, nonprotein nitrogen; PB_1_, true protein degraded rapidly; PB_2_, true protein degraded intermediately; PB_3_, true protein degraded slowly; PC, bound true protein; CP, crude protein. F-FTMR represents the FTMR based on fresh-alfalfa. E-FTMR represents the FTMR based on ensiled-alfalfa.

**Table 5:** The dynamics fermentation characteristics of fresh-alfalfa and ensiled-alfalfa FTMR after fermentation.

Item ^1^	Style	Additive	Fermentation Time, d						SEM	*p*-Value		
			1	3	7	15	30	60		S	A	S×A
pH	F-FTMR	CK	4.88	4.75 ^d^	4.70 ^b^	4.33 ^ab^	4.17 ^a^	4.22 ^a^	0.03	>0.05	>0.05	>0.05
		MOL	4.84	4.77 ^cd^	4.72 ^b^	4.37 ^a^	4.20 ^a^	4.14 ^ab^				
		LP	4.91	4.77 ^cd^	4.57 ^c^	4.14 ^d^	4.11 ^b^	4.14 ^ab^				
		MOL+LP	4.89	4.88 ^ab^	4.69 ^b^	4.23 ^c^	4.18 ^a^	4.14 ^ab^				
	E-FTMR	CK	4.87	4.94 ^a^	4.88 ^a^	4.36 ^ab^	4.07 ^bc^	4.11 ^bc^				
		MOL	5.00	4.88 ^ab^	4.99 ^a^	4.36 ^ab^	4.01 ^d^	4.02 ^c^				
		LP	4.88	4.83 ^bc^	4.89 ^a^	4.32 ^b^	4.01 ^d^	4.05 ^bc^				
		MOL+LP	4.93	4.88 ^ab^	4.91 ^a^	4.30 ^b^	4.03 ^cd^	4.04 ^c^				
LA (%DM)	F-FTMR	CK	0.67 ^b^	0.90 ^b^	1.39 ^cd^	3.25 ^bc^	4.17 ^b^	5.19 ^c^	0.09	<0.01	>0.05	>0.05
		MOL	0.89 ^b^	0.90 ^b^	1.16 ^d^	3.44 ^ab^	4.06 ^b^	5.10 ^c^				
		LP	0.82 ^b^	1.49 ^a^	1.52 ^bcd^	3.82 ^a^	3.88 ^b^	4.24 ^d^				
		MOL+LP	0.91 ^b^	1.73 ^a^	2.10 ^a^	2.72 ^cd^	4.12 ^b^	4.45 ^d^				
	E-FTMR	CK	1.66 ^a^	1.92 ^a^	1.83 ^abc^	2.58 ^d^	6.60 ^a^	8.38 ^a^				
		MOL	1.79 ^a^	1.49 ^a^	1.88 ^abc^	2.78 ^cd^	4.05 ^b^	8.22 ^a^				
		LP	1.81 ^a^	1.62 ^a^	1.93 ^ab^	2.62 ^d^	3.36 ^b^	7.65 ^b^				
		MOL+LP	2.00 ^a^	1.59 ^a^	1.88 ^abc^	2.76 ^cd^	4.10 ^b^	7.50 ^a^				
AA (%DM)	F-FTMR	CK	0.21	0.62 ^a^	0.61 ^abc^	0.70	1.05 ^ab^	1.73	0.02	<0.05	>0.05	>0.05
		MOL	0.51	0.65 ^a^	0.70 ^ab^	1.20	1.06 ^ab^	1.72				
		LP	0.28	0.65 ^a^	0.70 ^ab^	1.12	0.81 ^abc^	0.95				
		MOL+LP	0.38	0.88 ^a^	0.92 ^ab^	0.95	1.21 ^a^	1.46				
	E-FTMR	CK	0.34	0.28 ^b^	0.32 ^bc^	0.27	0.88 ^abc^	1.82				
		MOL	0.24	0.18 ^b^	0.24 ^c^	0.34	0.82 ^abc^	1.55				
		LP	0.38	0.21 ^b^	0.33 ^bc^	0.42	0.53 ^c^	1.61				
		MOL+LP	0.26	0.20 ^b^	0.26 ^bc^	0.47	0.71 ^bc^	1.63				
PA (%DM)	F-FTMR	CK	0.00 ^c^	0.15	0.24	0.41	0.80 ^ab^	1.32	0.02	>0.05	<0.05	>0.05
		MOL	0.33 ^ab^	0.33	0.33	0.99	0.88 ^ab^	1.66				
		LP	0.08 ^bc^	0.30	0.18	0.57	0.60 ^bc^	0.83				
		MOL+LP	0.19 ^bc^	0.53	0.59	0.75	1.11 ^a^	1.47				
	E-FTMR	CK	0.22 ^abc^	0.22	0.27	0.17	0.54 ^bc^	1.00				
		MOL	0.48 ^a^	0.36	0.51	0.35	0.68 ^bc^	1.38				
		LP	0.22 ^abc^	0.18	0.33	0.26	0.32 ^c^	0.96				
		MOL+LP	0.47 ^a^	0.30	0.40	0.39	0.58 ^bc^	1.27				
AN (%TN)	F-FTMR	CK	0.29 ^e^	0.49 ^d^	0.44 ^c^	1.90 ^bc^	3.67 ^d^	2.78 ^e^	0.22	<0.01	>0.05	>0.05
		MOL	0.18 ^f^	0.49 ^d^	0.44 ^c^	2.31 ^b^	2.92 ^e^	2.33 ^f^				
		LP	0.07 ^h^	0.53 ^d^	0.53 ^c^	1.09 ^bc^	2.40 ^e^	2.13 ^g^				
		MOL+LP	0.09 ^g^	0.45 ^d^	0.70 ^c^	0.75 ^c^	2.60 ^e^	1.55 ^h^				
	E-FTMR	CK	6.46 ^a^	7.27 ^a^	6.29 ^b^	5.76 ^a^	7.01 ^a^	6.74 ^b^				
		MOL	5.84 ^b^	6.63 ^bc^	6.46 ^ab^	5.14 ^a^	6.25 ^b^	6.27 ^c^				
		LP	5.19 ^d^	6.35 ^c^	6.15 ^b^	5.65 ^a^	5.28 ^c^	6.12 ^d^				
		MOL+LP	5.61 ^c^	6.90 ^ab^	6.98 ^a^	5.57 ^a^	5.48 ^c^	6.96 ^a^				

^1^ LA, lactic acid; AA, acetic acid; PA, propionic acid; and AN, ammonia nitrogen. F-FTMR represents the FTMR based on fresh-alfalfa. E-FTMR represents the FTMR based on ensiled-alfalfa. MOL, LP and MOL+LP represent the addition of molasses, *Lactobacillus plantarum* and molasses mixed with *L. plantarum*, respectively. Moreover, CK represents the addition of an equal amount of distilled water to the diets. DM represents dry matter and TN represents total nitrogen. A denotes significance of the additive, S denotes significance of two fermented style of FTMR, and A×S denotes significance of the interaction between the additive and style. SEM, standard error of means. ^a–h^ Means within a column with no common superscript differ (*p* < 0.05).

**Table 6:** Statistical analysis for the fermentation profile variables of fresh-alfalfa and ensiled-alfalfa FTMR treated with different additives at each ensiling day.

Item ^1^	Fermentation Time, d					
	1	3	7	15	30	60
pH	>0.05	<0.01	<0.01	<0.01	<0.01	<0.01
LA	<0.01	<0.01	<0.05	<0.01	<0.01	<0.01
AA	>0.05	<0.01	<0.05	>0.05	<0.05	>0.05
PA	<0.01	>0.05	>0.05	>0.05	<0.01	>0.05
AN	<0.01	<0.01	<0.01	<0.01	<0.01	<0.01

^1^ LA, lactic acid; AA, acetic acid; PA, propionic acid; and AN, ammonia nitrogen.

**Table 7:** The chemical compositions of fresh-alfalfa and ensiled-alfalfa FTMR after 60 d of fermentation.

Item ^1^	F-FTMR				E-FTMR				SEM	*p*-Value		
	CK	MOL	LP	MOL+LP	CK	MOL	LP	MOL+LP		S	A	S×A
DM (%)	36.02 ^d^	36.72 ^cd^	36.71 ^cd^	37.19 ^c^	47.60 ^b^	49.20 ^a^	49.53 ^a^	48.64 ^a^	1.80	<0.01	<0.05	>0.05
CP (%DM)	12.38 ^a^	12.65 ^a^	12.38 ^a^	12.62 ^a^	11.38 ^b^	10.92 ^b^	10.92 ^b^	11.16 ^b^	0.22	<0.01	>0.05	>0.05
aNDF (%DM)	34.44 ^b^	33.39 ^b^	35.53 ^ab^	34.70 ^ab^	33.74 ^b^	30.64 ^c^	33.12 ^b^	36.83 ^a^	0.59	>0.05	<0.01	<0.05
ADF (%DM)	21.85	21.18	20.86	24.62	21.90	20.83	19.67	22.87	0.60	>0.05	<0.05	>0.05
WSC (%DM)	0.13 ^bc^	0.15 ^bc^	0.11 ^bc^	0.19 ^b^	0.05 ^c^	0.32 ^a^	0.10 ^bc^	0.16 ^bc^	0.03	>0.05	<0.01	<0.05
TDN (%DM)	71.64	72.17	72.42	69.45	71.60	72.44	73.36	70.83	0.47	>0.05	<0.05	>0.05
RFV	194.19 ^bcd^	201.97 ^bc^	190.20 ^bcd^	186.94 ^cd^	198.73 ^bc^	221.03 ^a^	207.07 ^ab^	179.73 ^d^	4.35	>0.05	<0.01	>0.05

^1^ F-FTMR represents the FTMR based on fresh-alfalfa. E-FTMR represents the FTMR based on ensiled-alfalfa. MOL, LP, and MOL+LP represent the addition of molasses, *Lactobacillus plantarum* and molasses mixed with *L. plantarum*, respectively. CK represents the addition of an equal amount of distilled water to the diets. SEM, standard error of means. A denotes significance of the additive, S denotes significance of two fermented style of FTMR, and A×S denotes significance of the interaction between the additive and style. DM, dry matter; CP, crude protein; aNDF, neutral detergent fiber; ADF, acid detergent fiber; WSC, water-soluble carbohydrates; TDN, total digestible nutrient; and RFV, relative feeding value. ^a–d^ Means within a raw with no common superscript differ (*p* < 0.05).

## Data Availability

The data presented in this study are available on request from the corresponding author.
